# Genome-wide identification and expression characterization of the *GH3* gene family of tea plant (*Camellia sinensis*)

**DOI:** 10.1186/s12864-024-10004-y

**Published:** 2024-01-27

**Authors:** Xinge Wang, Chunyu Jia, Lishuang An, Jiangyan Zeng, Aixia Ren, Xin Han, Yiqing Wang, Shuang Wu

**Affiliations:** 1 School of Life Science and Agriculture, Qiannan Normal University for Nationalities, Duyun, Guizhou 558000 China; 2 Key Laboratory of Green Pesticide and Agricultural Bioengineering, Ministry of Education, Guizhou University, Guiyang, Guizhou 550025 China

**Keywords:** *Camellia sinensis*, GH3 gene family, Gene expression analysis, Transcriptional regulation, Genome-wide identification

## Abstract

To comprehensively understand the characteristics of the *GH3* gene family in tea plants (*Camellia sinensis*), we identified 17 *CsGH3* genes and analyzed their physicochemical properties, phylogenetic relationships, gene structures, promoters, and expression patterns in different tissues. The study showed that the 17 *CsGH3* genes are distributed on 9 chromosomes, and based on evolutionary analysis, the *CsGH3* members were divided into three subgroups. Gene duplication analysis revealed that segmental duplications have a significant impact on the amplification of *CsGH3* genes. In addition, we identified and classified *cis*-elements in the *CsGH3* gene promoters and detected elements related to plant hormone responses and non-biotic stress responses. Through expression pattern analysis, we observed tissue-specific expression of *CsGH3.3* and *CsGH3.10* in flower buds and roots. Moreover, based on predictive analysis of upstream regulatory transcription factors of *CsGH3*, we identified the potential transcriptional regulatory role of gibberellin response factor CsDELLA in *CsGH3.14* and *CsGH3.15*. In this study, we found that *CsGH3* genes are involved in a wide range of activities, such as growth and development, stress response, and transcription. This is the first report on *CsGH3* genes and their potential roles in tea plants. In conclusion, these results provide a theoretical basis for elucidating the role of *GH3* genes in the development of perennial woody plants and offer new insights into the synergistic effects of multiple hormones on plant growth and development in tea plants.

**Supplementary Information:**

The online version contains supplementary material available at 10.1186/s12864-024-10004-y.

## Background

Tea plant (*Camellia sinensis* (L.) O. Kuntze) is an extremely important economic crop in the world, widely loved by consumers due to its good health benefits, such as weight loss, blood glucose reduction, antioxidant properties [1, 2], and alleviating hypercholesterolemia [3, 4]. Tea plant leaves are the main processing material [5], and the formation of plant leaf morphological characteristics is usually a very complex physiological and biochemical mechanism [6]. Understanding the mechanism and mechanism of tea leaf growth and development is of great significance for the picking, storage, and processing of tea leaves, and can also increase their economic value. Plant hormones play a vital role in the growth and development of plant leaves [7–9], including controlling the growth and differentiation of leaf primordia [10], controlling the differentiation of vasculature [11], cell division of leaves [12], and cell expansion of leaves [13].

Plant hormones include auxins, gibberellins, cytokinins, abscisic acid, ethylene, brassinosteroids, etc. Among them, auxin, as one of the important hormones in the plant life cycle, regulates cell division, elongation, and differentiation [14, 15]. It mainly controls plant growth and development by regulating the expression of the auxin-responsive gene families, including GH3, SAURs, AUX/IAA, and ARF [16, 17]. GH3 family proteins play important roles in the regulation of plant hormone homeostasis and signaling pathways [18]. The IAA-amido synthetase encoded by the GH3 gene family catalyzes the conjugation of auxin and the binding of free IAA to amino acids to maintain auxin homeostasis [19, 20]. GH3 proteins, as enzymes, are also involved in the synthesis of IAA and JA [21], and they possess amino acid synthetase activity. By regulating hormone and stress-related signaling pathways, they bind excess IAA, SA, and JAS to amino acids to maintain hormone homeostasis [22–24]. In addition, some *GH3* genes are associated with developmental regulation and stress response. Overexpression of *GH3* can enhance plant disease resistance by inhibiting cell wall loosening and reducing auxin levels [25]. *GH3* can also reduce endogenous auxin levels to enhance plant drought tolerance [26] and regulate ABA levels to influence plant drought and cold resistance [27].

Currently, the GH3 family has been identified in many plant species. For example, 19 family members in *Arabidopsis thaliana* (L.) Heynh [28], 13 in rice (*Oryza sativa*) [29], 12 in maize [30], 15 in tomato (*Solanum lycopersicum*) [31], 2 in longan (*Dimocarpus longan* L.) [32], 9 in grape (*Vitis vinifera* L.) [33], 15 in apple (*Malus × domestica*) [24], and 11 in citrus (*Citrus sinensis* L.) [34]. Although *GH3* has been identified in these species, little is known about the GH3 gene family in tea plants, and its evolution, function, and classification in tea plants have not been systematically studied. The identification of the GH3 gene family in tea plants will be important for the picking, storage, and processing of tea leaves. Therefore, based on the genomic data of tea plants, this study aims to identify the GH3 family members and analyze their chromosomal localization, collinearity analysis, evolutionary relationships, promoter *cis*-acting elements, codon preferences, etc. The GH3 gene family will be validated using qRT-PCR and yeast one-hybrid techniques. The results of this study will provide insights into the function of the GH3 gene family in tea plants and lay a theoretical foundation for further research on tea leaf growth and development.

## Materials and methods

### Plant materials

The tea plant variety selected for this experiment is ‘Fuding Dabai’ (Plants were cultivated in the greenhouse of Guizhou University East Campus in Guiyang at a room temperature of 25 °C and a light cycle of 16 h/20°C.). In May 2023, well-grown and healthy shoots with one bud and two leaves were harvested. After sampling, the plant materials were snap-frozen in liquid nitrogen and stored at -80℃ for future use. Each treatment was replicated three times for biological analysis.

### Identification and characteristic analysis of *CsGH3* gene family members

The complete genome sequence, proteome data, and genome annotation files of the ‘Tieguanyin’ tea plant were downloaded from the Tea Plant Information Archive (TPIA): A comprehensive knowledge database for tea plants (teaplants.cn) [35]. The hidden Markov model (PF03321) of GH3 from the Pfam database (http://pfam.Xfam.org/) was used to screen the protein sequences of the tea plant [36]. The amino acid sequences of GH3 gene family in *Arabidopsis thaliana* were used as a reference to retrieve homologous genes in the tea plant through BLAST in TBtools (https://github.com/CJ-Chen/TBtools) [37]. The results of the two search methods were combined, redundant sequences were removed, and the amino acid sequences of candidate members were submitted to the NCBI’s CDD database (https://www.ncbi.nlm.nih.gov/cdd/) to manually delete members with incomplete N/C terminals [38]. Finally, the GH3 gene family members of the ‘Tieguanyin’ tea plant were obtained.

The protein physicochemical properties of CsGH3 were analyzed using the online website ExPASy (https://web.expasy.org/protparam) [39], and subcellular localization prediction of CsGH3 was conducted using the online website wolf-psort (https://www.genscript.com/wolf-psort.html) [40].

### Construction of the CsGH3 phylogenetic tree

The GH3 protein sequences of the tea plant, *Arabidopsis thaliana*, maize (*Zea mays*), and woodland strawberry (*Fragaria vesca* L.) were aligned using MEGA 7.0 [41]. The neighbor-joining method was used to construct the phylogenetic tree of the GH3, with a bootstrap value set to 1000 and other parameters set to default values [42].

### Chromosomal localization and collinearity analysis of *CsGH3* gene family

The chromosome location information of CsGH3 gene family members was extracted from the tea plant genome gff file using the TBtools [43] software, and visualizations were generated.

The TBtools software’s Advanced Circos function was utilized to perform intraspecific and interspecific collinearity analysis of the CsGH3 gene family.

### Analysis of *CsGH3* conserved motif, gene structure, promoter *Cis*-acting element, and codon preference

The full-length protein sequences of the identified *CsGH3* genes were subjected to conservation sequence analysis, identification of important functional sites, and motif analysis using the online software MEME (https://meme-suite.org/meme/tools/meme) with default parameters [44]. The gene structures of *CsGH3* were visualized and analyzed using the TBtools software. The upstream 2000 bp sequences of CsGH3 gene family members were extracted using the TBtools software [45, 46]. Plant CARE (http://bioinformatics.psb.ugent.be/webtools/plantcare/html/) online software was employed to predict *cis*-acting elements in these sequences [47]. Codon W 1.4.4 software (https://codonw.sourceforge.net/culong.html#CodonW) were utilized to obtain the major parameters of codons and the relative synonymous codon usage (RSCU) for the tea plant GH3 gene family members.

### Prediction and validation of upstream regulatory transcription factors of *CsGH3*

The upstream 2000 bp sequences of CsGH3 gene family members were submitted to the PlantTFDB database Regulation Prediction (http://plantregmap.gao-lab.org/regulation_prediction.php) [48]. The submission criteria were set as p-value ≤ e-7, with *Arabidopsis thaliana* as the reference species, to predict the upstream regulatory transcription factors of *CsGH3*. The tea plant gene sequence IDs were obtained by performing BLASTP in the TPIA database against *Arabidopsis thaliana* homologous genes.

Selected predicted transcription factors such as CsDELLA, CsSOC, CsBBR, and CsREM, together with *CsGH3.14Pro* and *CsGH3.15Pro*, were validated using yeast one-hybrid (Y1H) assay. Primers were designed based on the gene promoter and coding region sequences (Table S1). The *Hind III* and *Xho I* restriction sites were used for pAbAi vector digestion, while the *EcoR I* and *BamH I* restriction sites were used for pGADT7 vector digestion. All the required recombinant vectors were constructed using the ClonExpress II One Step Cloning Kit (Nanjing Novogene Bioinformatics Technology Co., Ltd.).

The recombinant vectors pAbAi-P53, pAbAi-*CsGH3.14*Pro, and pAbAi-*CsGH3.15*Pro were digested with *BstB I* and transformed into yeast Y1H competent cells using the PEG/LiAc method. To screen for the appropriate concentration of aureobasidin A (AbA) that inhibits the growth of Y1H(*CsGH3.14*Pro) and Y1H(*CsGH3.15*Pro), pAbAi-P53 was used as a positive control. The transformed cells were cultured on SD/-URA plates containing different concentrations of AbA (0, 50, 150, 300, 500 ng·mL^− 1^) for 3–5 days, and colony formation was observed. After determining the inhibitory concentration of AbA, the one-step yeast transformation kit (Wuhan Puner Biotechnology Co., Ltd.) was used to transform pGADT7 and the corresponding pGADT7-DELLA (TGY052266), pGADT7-SOC (TGY105712), pGADT7-BBR (TGY100531), pGADT7-REM (TGY021590) into Y1H(*CsGH3.14*Pro) and Y1H(*CsGH3.15*Pro) competent cells. This resulted in yeast recombinant strains, including Y1H(*CsGH3.14*Pro + pGADT7), Y1H(*CsGH3.14*Pro + CsDELLA), Y1H(*CsGH3.14*Pro + CsBBR), Y1H(*CsGH3.14*Pro + CsSOC), Y1H(*CsGH3.15*Pro + pGADT7), Y1H(*CsGH3.15*Pro + CsDELLA), Y1H(*CsGH3.15*Pro + CsSOC), and Y1H(*CsGH3.15*Pro + CsREM). The strains were cultured on SD/-Leu and SD/-Leu/AbA50 plates for 3–5 days. Y1H(*CsGH3.14*Pro + pGADT7) and Y1H(*CsGH3.15*Pro + pGADT7) strains were used as negative controls, and colony formation was observed.

## Results

### Identification and physicochemical analysis of the *CsGH3* gene family

Through HMMER and BLAST searches, a total of 17 *GH3* genes were identified in the ‘Tieguanyin’ tea plant genome. Based on their chromosomal distribution, they were named *CsGH3.1*-*CsGH3.17*. The molecular weights ranging from 53.7 kDa to 73.7 kDa. The isoelectric points (pI) of these gene family members were all below 7, indicating that they are acidic hydrophilic proteins. The hydrophilicities were all below 0, indicating a strong hydrophilic nature. The protein instability index values were all below 40, suggesting that they are stable proteins. Among them, CsGH3.8, CsGH3.11, CsGH3.12, and CsGH3.16 were identified as stable proteins, while the rest were classified as unstable proteins. Subcellular localization prediction results showed that the 17 GH3 proteins were distributed widely in the cell nucleus, cytoplasm, and chloroplasts (Table S2).

**Fig. 1:**
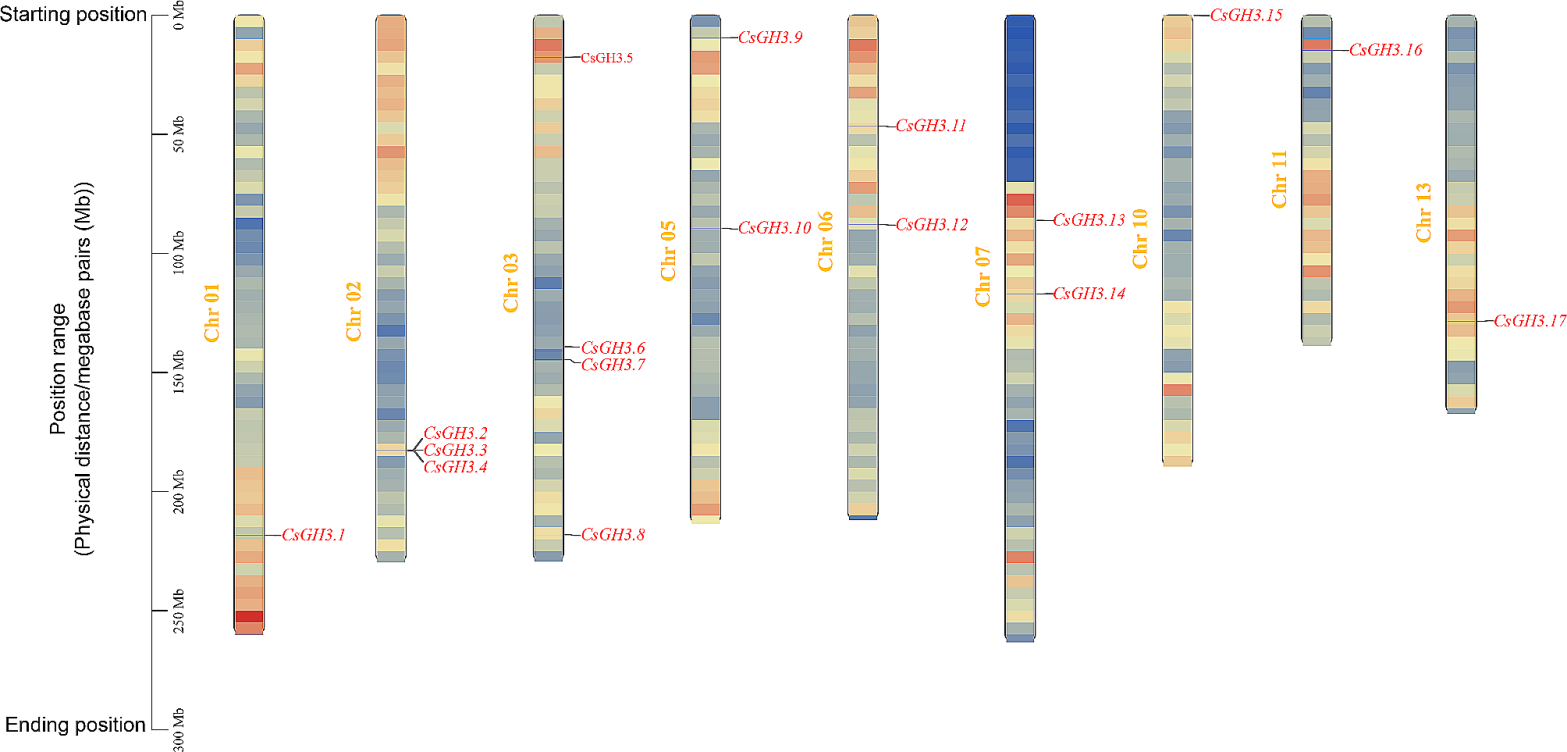
The distribution of 17 *CsGH3* genes on the chromosomes. 0 Mb to 300 Mb represents the position range of gene family on a chromosome. This range is measured in physical distance on the chromosome and is typically expressed in megabase pairs (Mb). Specifically, 0 Mb represents the starting position of the gene, while 300 Mb represents the ending position

The 17 *CsGH3* genes were distributed on 9 chromosomes (Chr 01, Chr 02, Chr 03, Chr 05, Chr 06, Chr 07, Chr 10, Chr 11, Chr 13). Among these chromosomes, Chr 03 had the highest number of *CsGH3* genes with 4 members, including *CsGH3.5*, *CsGH3.6*, *CsGH3.7*, and *CsGH3.8*. Following that, Chr 02 had 3 members, while Chr 05, Chr 06, and Chr 07 each had 2 members. The remaining chromosomes each had only 1 *CsGH3* gene (Fig. 1).

### Collinearity analysis of the *CsGH3* gene family

**Fig. 2:**
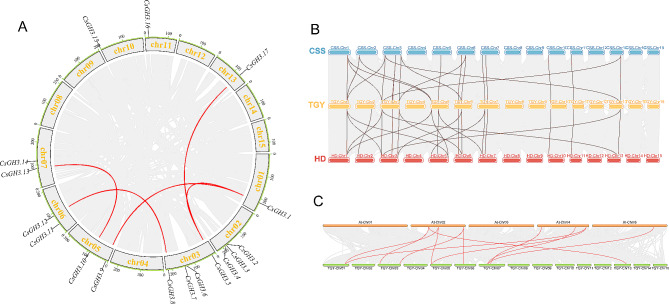
Collinearity Analysis of the Tea Plant GH3 Gene Family. (**A**) Chromosomal distribution of *CsGH3 *genes in tea plants. (**B**) Colinearity analysis of *CsGH3* in three tea plant varieties, included Shuchazao (CSS), Tieguanyin (TGY), and Huangdan (HD). (**C**) Colinearity analysis of *CsGH3* between Tieguanyin of tea plants and Arabidopsis

Gene duplication events play an important role in the amplification of gene family members during plant evolution. Intraspecific collinearity analysis revealed the presence of 5 pairs of collinear genes in the ‘Tieguanyin’ tea plant (*CsGH3.1*-*CsGH3.5*, *CsGH3.1*-*CsGH3.9*, *CsGH3.5*-*CsGH3.17*, *CsGH3.8*-*CsGH3.12*, *CsGH3.10*-*CsGH3.13*), all of which were attributed to segmental duplication (Fig. 2A). No tandem duplication events were identified, indicating that segmental duplication was the primary mode of expansion for the CsGH3 gene family.

The collinearity analysis between ‘Tieguanyin’ and ‘Shuchazao’ and ‘Huangdan’ tea plant varieties revealed the presence of 22 pairs and 24 pairs of paralogous genes, respectively, indicating a high degree of conservation in the evolution of the CsGH3 gene family (Fig. 2B).

In the collinearity analysis between the ‘Tieguanyin’ tea plant and the *Arabidopsis thaliana* genome, it was found that there were 11 pairs of collinear *GH3* genes between the two species (Fig. 2C, Table S4). It was observed that a single gene had multiple collinear counterparts, such as *CsGH3.1*-*AtGH3.2*/*AtGH3.3*, *CsGH3.9*-*AtGH3.2*/*AtGH3.3*, and *CsGH3.13*-*AtGH3.5*/*AtGH3.6*. This indicates the conservation of duplicated genes and the existence of ancient gene pairs before the divergence of *Arabidopsis thaliana* and the tea plant. The high conservation between GH3 proteins of different species suggests the functional similarity between the GH3 proteins of the tea plant and those of other species.

### GH3 evolutionary analysis in tea plants

**Fig. 3:**
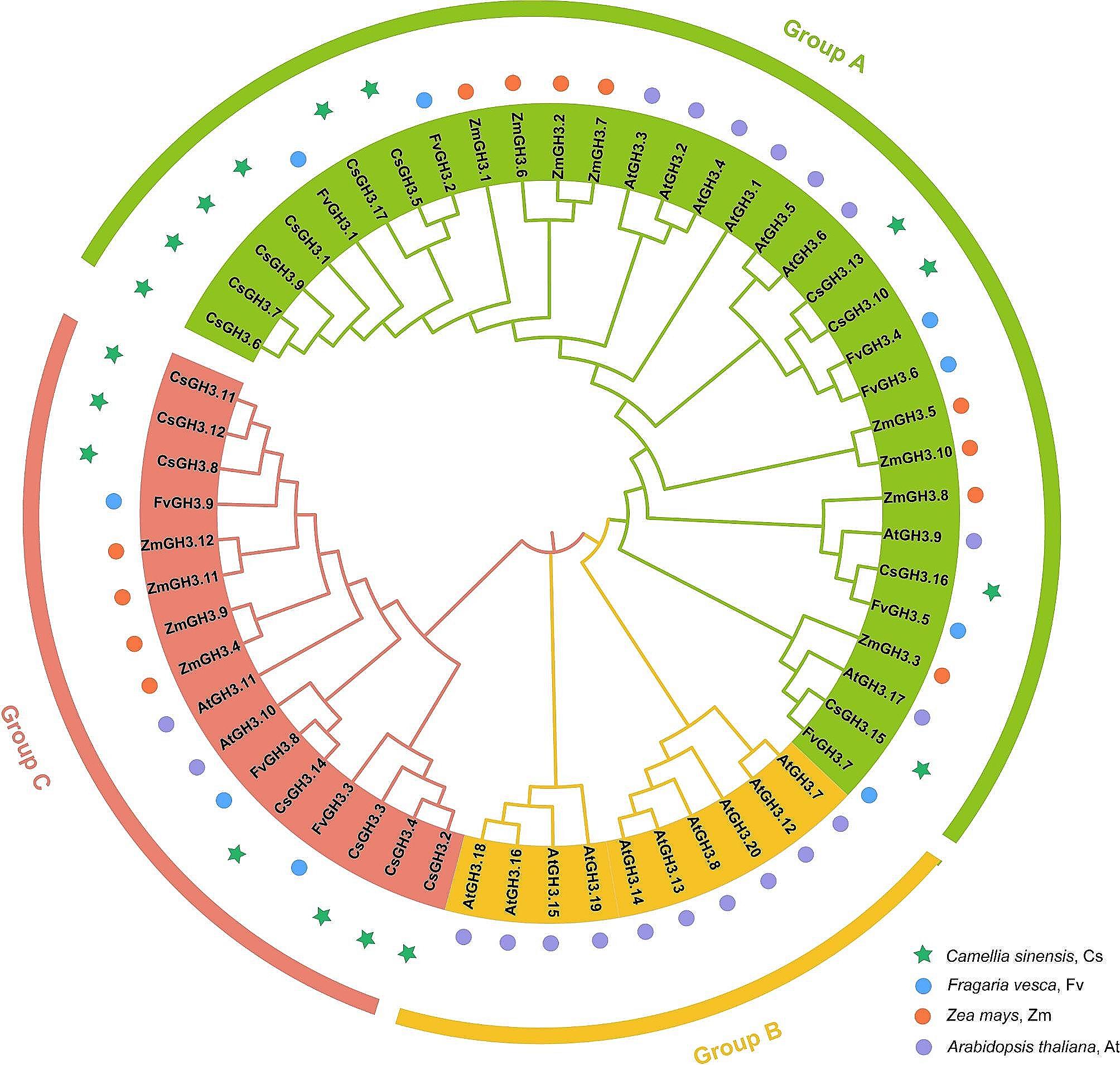
Phylogenetic tree of the GH3 family proteins in *Camellia sinensis*, *Fragaria vesca*, *Zea mays*, and *Arabidopsis thaliana*

In order to deeply understand the evolutionary patterns of GH3 in different species, the MEGAX software was used to perform multiple sequence alignment on 58 GH3 proteins from four species: tea plants (*Camellia sinensis*), *Arabidopsis thaliana*, maize (*Zea mays*), and woodland strawberry (*Fragaria vesca*) (Fig. 3). The results showed that all GH3 proteins were mainly classified into three groups: Group A, Group B, and Group C. Among them, Group B did not contain any distribution of CsGH3 members from tea plants but only included AtGH3 members from Arabidopsis. Group A contained 10 CsGH3 members, and Group C contained 7 members. In addition, the affinity between GH3 proteins of tea plants and woodland strawberry was the closest, indicating a certain functional similarity between GH3 proteins in tea plants and woodland strawberry.

### Codon usage bias and selection pressure analysis in *CsGH3* gene family

The analysis of codon usage bias in the CsGH3 gene family revealed that the GC content ranged from 0.417 to 0.519, with an average of 0.47. The frequency of G or C in the third codon position (GC3s) ranged from 0.343 to 0.606, with a mean of 0.49. The frequencies of A, T, G, and C in the third codon position (A3s, T3s, C3s, G3s) were 0.29, 0.34, 0.33, and 0.30, respectively.

The effective number of codons (ENc) reflects the degree of codon usage bias in genes, and it ranged from 21 to 60. The closer the ENc value is to 20, the stronger the codon preference. The mean ENc value for the CsGH3 gene family was 55.28. The codon adaptation index (CAI) is another important parameter for measuring codon usage bias, and the mean CAI value for the CsGH3 gene family was 0.21. Overall, the CsGH3 gene family showed a weak codon usage bias, and the gene expression level was relatively low.

The Ka/Ks ratio can be used to determine whether there is selection pressure on the protein-coding genes. It plays an important role in the evolutionary analysis of gene families. The Ka/Ks analysis was performed on *CsGH3 genes* (Table S3). The results showed that the Ka/Ks values for gene duplication events ranged from 0.079 to 0.140. All duplicated genes had Ka/Ks values less than 1, indicating that these genes evolved under purifying selection pressure.

### Gene structure and conserved motif analysis of the tea plant *GH3* gene family

**Fig. 4:**
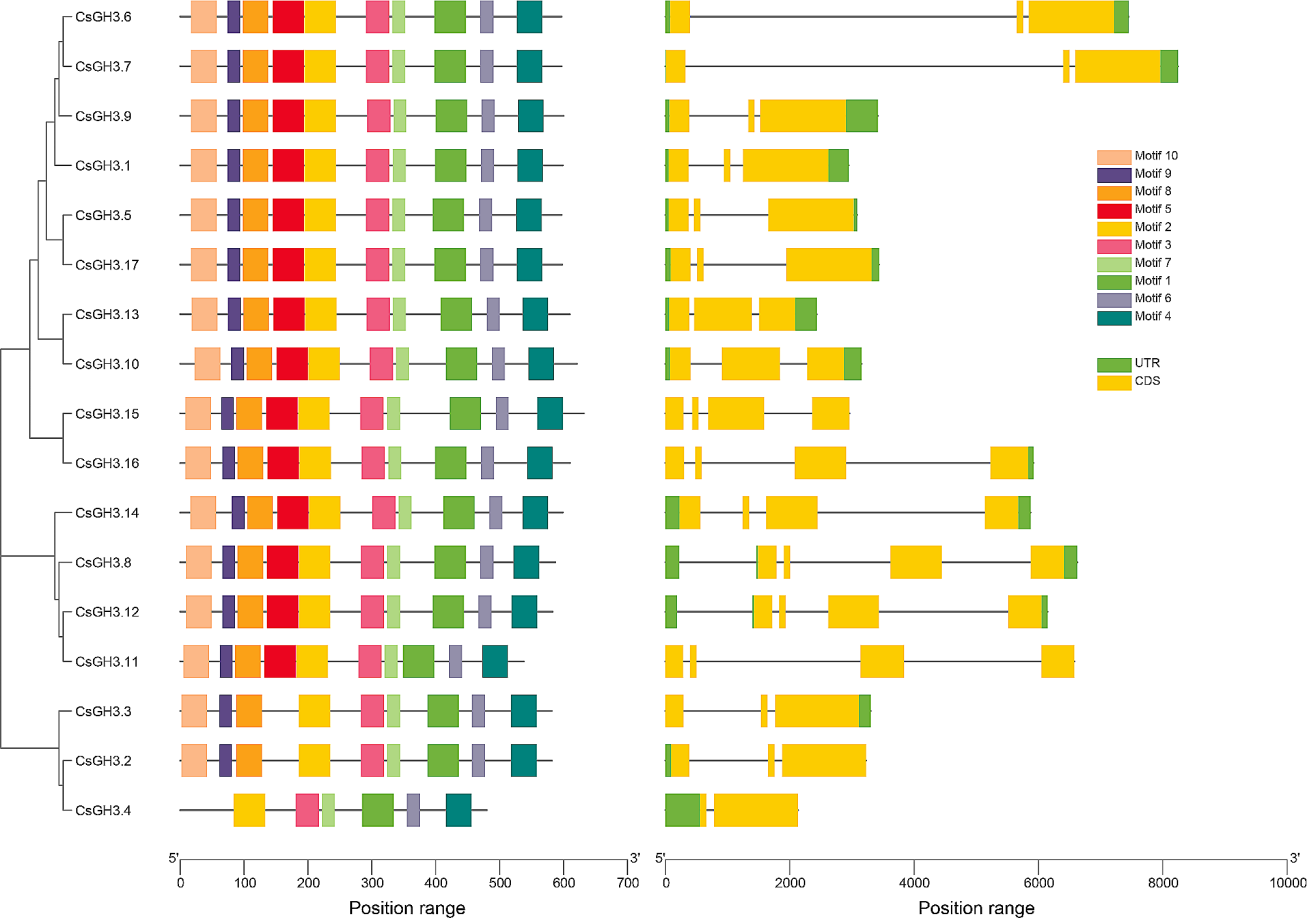
Phylogenetic tree, core motif, and protein domain of tea plants GH3 proteins. The scales in motif and domain analyses represent the relative positions of specific motifs or different domains, respectively

The number of exons in *CsGH3* gene members ranges from 2 to 4, with most members consisting of 3 exons. The analysis of conserved motifs showed that the number of conserved motifs in most *CsGH3* gene members was consistent (Fig. 4). *CsGH3.4* only had 6 conserved motifs, lacking motifs 10, 9, 8, and 5. *CsGH3.2* and *CsGH3.3* contained 9 conserved motifs, including motif 5.

### Analysis of *Cis*-acting elements in the promoter region of the *CsGH3* gene family

**Fig. 5:**
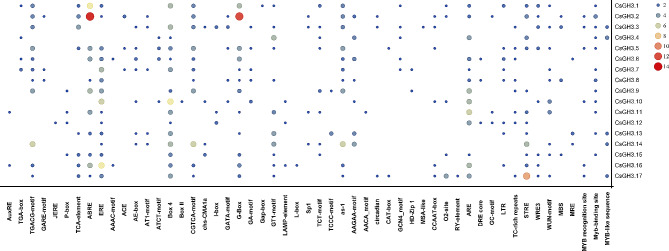
Analysis of *cis*-acting elements in the promoter region of the CsGH3 gene family. The dots, from small to large and from blue to red, represent increasing quantity

The TBtools software was used to extract the 2000 bp upstream gene sequence of the *CsGH3* gene as the promoter sequence of *CsGH3*. The results showed that the *cis*-acting elements of the 17 *CsGH3* members can be divided into five categories: phytohormone-responsive elements, light-responsive elements, plant growth and development-related elements, abiotic stress-responsive elements, and transcription factor recognition and binding sites (Fig. 5). Among them, light-responsive elements accounted for the highest proportion, followed by phytohormone-responsive elements. Phytohormone-responsive elements include auxin-responsive elements (AuxRE, TGA-box) and gibberellin-responsive elements (GARE-motif, P-box). The promoter regions of *CsGH3.1* and *CsGH3.2* respectively contain 7 and 13 abscisic acid responsiveness elements (ABRE), suggesting their main involvement in ABA response. In addition, anaerobic induction responsive elements are an important part of the stress-responsive elements, and *CsGH3.9*, *CsGH3.10*, *CsGH3.11*, and *CsGH3.16* contain a higher number of anaerobic induction responsive elements, suggesting their important roles in plant anaerobic response. In conclusion, *CsGH3* not only performs normal transcription activities, but also participates in plant light response, hormone response, stress response, and growth and development activities.

### qRT-PCR analysis of the *CsGH3* gene family

**Fig. 6:**
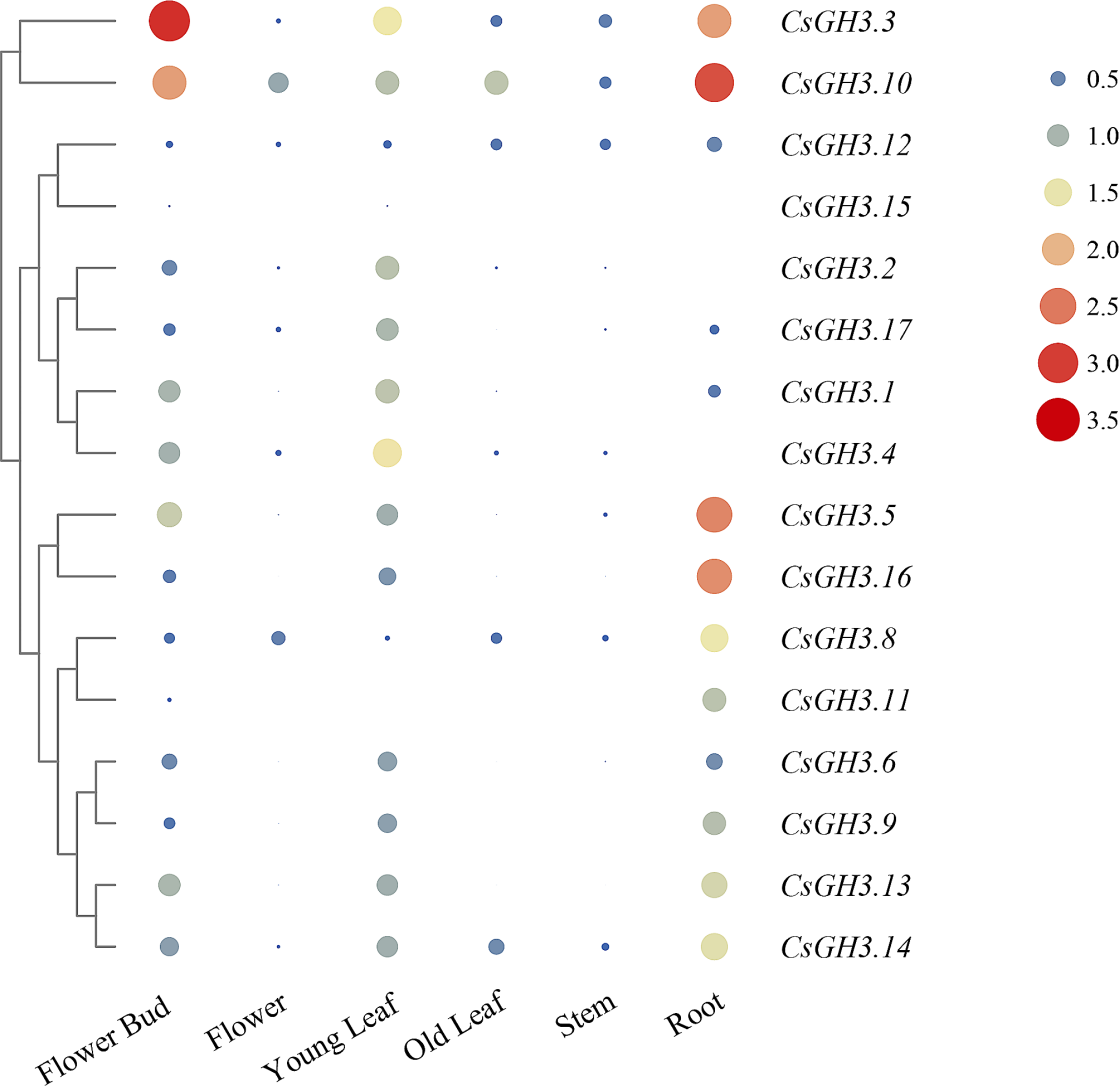
qRT-PCR analysis of the CsGH3 gene family. The dots, from small to large and from blue to red, represent increasing abundance

The qRT-PCR analysis was performed to investigate the expression patterns of the CsGH3 gene family. Total RNA was extracted from various tissues including leaves, stems, roots, and flowers. Reverse transcription was carried out to synthesize cDNA, and qPCR was conducted to quantify the expression levels of the *CsGH3* genes. The results showed differential expression patterns among the CsGH3 gene family members in different tissues (Fig. 6). Most *GH3* genes are expressed in different tissues, while *CsGH3.15* is expressed at a low level in various tissue parts. Among the gene family members, four genes (*CsGH3.3*, *CsGH3.10*, *CsGH3.5*, *CsGH3.16*) are highly expressed in roots, suggesting their potentially important role in tea tree roots. *CsGH3.3* and *CsGH3.10* are expressed higher in flower buds compared to other tissues, indicating their involvement in flower bud differentiation.

### Prediction and validation of upstream regulatory factors of the *CsGH3* gene family

**Fig. 7:**
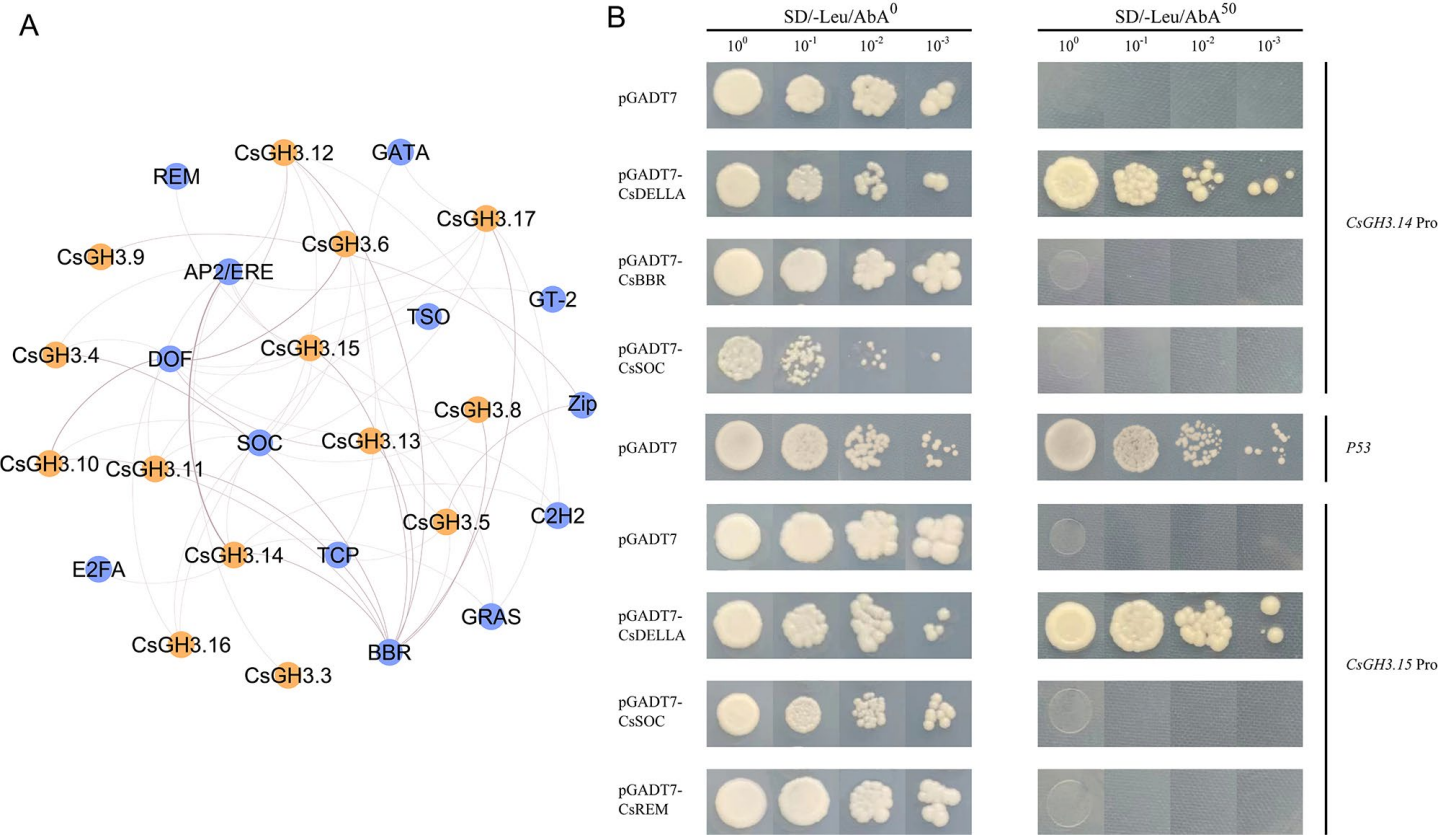
Prediction (**A**) and validation (**B**) of upstream regulatory factors of the CsGH3 gene family

Using the model plant *Arabidopsis thaliana* as a reference, transcription factors that potentially bind to the promoter regions of the CsGH3 gene family were predicted through the PlantTFDB website, followed by homology comparison in the tea plant. A total of 32 transcription factors were identified, mainly including AP2/ERF, DOF, ZIP, and other transcription factors (Fig. 7A, Tables S6 and S8).

The predicted results of transcriptional regulation were validated in vitro. Yeast one-hybrid (Y1H) assays showed that both pAbAi + *CsGH3.14*Pro and pAbAi + *CsGH3.15*Pro strains could grow normally on SD/-URA medium. By screening with different concentrations of AbA, it was found that both pAbAi + *CsGH3.14*Pro and pAbAi + *CsGH3.15*Pro strains stopped growing at a concentration of 50 ng·mL^− 1^ AbA. Therefore, the self-activation of *CsGH3.14*Pro and *CsGH3.15*Pro occurred at a concentration of 50 ng·mL^− 1^ AbA (Fig. 7B). Furthermore, the predicted CsDELLA (CsGRAS), CsSOC, CsBBR, and CsREM transcription factors were tested for their interaction with *CsGH3.14*Pro and *CsGH3.15*Pro in the yeast one-hybrid assay. The results showed that all yeast strains co-transformed with the bait and pGADT7 plasmids could grow normally on the SD/-Leu medium. On SD/-Leu medium supplemented with 50 ng·mL^− 1^ AbA, the *CsGH3.14*Pro + CsDELLA and *CsGH3.15*Pro + CsDELLA strains could grow, indicating the interaction between the CsDELLA transcription factor and *CsGH3.14*Pro, *CsGH3.15*Pro promoters.

## Discussion

The GH3 gene family was first discovered and named by Gretchen Hagen in soybean (*Glycine max*). Currently, there have been reports of the genome-wide identification of the GH3 gene family, not only in model plants such as Arabidopsis [28] and tobacco, but also in various other plant species, including cucumber [49], cabbage [50], walnut [51], and kiwifruit [52]. Indole-3-acetic acid (IAA) is of crucial importance for plant growth and development. Research has shown that GH3 proteins conjugate IAA with amino acids such as leucine, alanine, and phenylalanine to form conjugated IAA, serving as a storage form of IAA [19, 53]. This process is reversible and is a vital prerequisite for maintaining the homeostasis of plant growth hormone [54–56]. GH3 family proteins can catalyze the conjugation of jasmonic acid, salicylic acid, and other small molecule substrates (such as amino acids) to form conjugated hormones, thereby participating in hormone regulation in plants. Moreover, GH3 proteins are involved in plant stress responses. Silencing the expression of six *GH3* genes in apple has been found to contribute to better drought tolerance in transgenic plants, enhancing their adaptability to prolonged drought conditions [57]. *GH3* genes play a role not only in plant growth and development processes but also in influencing plant resistance. Therefore, the study of *GH3* genes holds significant importance in plant research. In this study, using bioinformatics methods, 17 *GH3* genes were identified in ‘Tieguanyin’ tea plants and named as *CsGH3.1*-*CsGH3.17*. They all had isoelectric points below 7. Bioinformatics analysis indicated that *CsGH3* genes exhibited high similarities in terms of amino acid sequences, gene structures, and conserved motifs, suggesting that although there are certain differences among the members of the GH3 gene family, they remain relatively conserved during evolutionary processes. This implies the presence of both functional similarity and differentiation among *CsGH3* genes, indicating their synergistic roles in regulating plant growth and development.

Based on protein sequence homology and substrate specificity differences, GH3 members in plants can be classified into three groups, mainly catalyzing the conjugation of JA, IAA, and SA with amino acids. The evolutionary tree results show that Group C contains AtGH3.10/DWARF IN LIGHT2 (DFL2) and AtGH3.11/AtJAR1. These two genes have been proven to be mainly involved in the biosynthesis of JA-amino acids [21, 58, 59]. The functionalities of Group B members have not been fully confirmed, and they have only been found in Arabidopsis and cruciferous plants [60–62]. Only *AtGH3.12* has been discovered to participate in the SA signaling pathway. In Group B, genes play important roles in IAA adenylation or amino acid conjugation reactions [19, 63]. Studies have found that members of Group A in Arabidopsis have undergone gene duplication events [61]. These newly duplicated members are mostly redundant in Arabidopsis. Based on the distribution of *GH3* members in different evolutionary branches and collinearity analysis in tea plants, *GH3* members in tea plants are also enriched in Group A. Gene fragment duplication events have occurred four times in Group A: *CsGH3.1*-*CsGH3.5*, *CsGH3.1*-*CsGH3.9*, *CsGH3.5*-*CsGH3.17*, *CsGH3.10*-*CsGH3.13*. Collinearity analysis between Arabidopsis and ‘Tieguanyin’ tea plants shows that gene family expansion has led to more collinear genes between Arabidopsis and tea plants. Based on the results of intra-species and inter-species collinearity, it is inferred that the gene duplication events of *CsGH3.5* and *CsGH3.1* occurred earlier and underwent more evolution, resulting in different collinear genes compared to Arabidopsis. After continuous evolution and selection in tea plants, *CsGH3.5* and *CsGH3.1* were duplicated into *CsGH3.17* and *CsGH3.9*, respectively. Therefore, the collinear genes of *CsGH3.1*-*CsGH3.9* and *CsGH3.5*-*CsGH3.17* are consistent with Arabidopsis.

Through the analysis of gene promoter regions, it is beneficial to understand and predict the potential *cis*-acting elements that they may contain, which is of great significance for further research on gene function. In this study, the upstream 2000 bp sequence of *CsGH3* was analyzed, and it was found that the promoter region of the *CsGH3* gene contains various elements related to hormone response and light response. Light is one of the necessary conditions for plant growth and development, and as a key factor in auxin response, auxin-responsive elements (AuxRE, TGA-box) were found in the promoter regions of genes *CsGH3.1*, *CsGH3.4*, *CsGH3.6*, *CsGH3.7*, *CsGH3.11*, and *CsGH3.16*. SA response elements were only found in *CsGH3.12*, and *CsGH3.11* only contained six elements related to ethylene response. Additionally, a significant number of ABA response elements (ABRE) were found in the upstream regions of most members (11 genes). This suggests that the function of the CsGH3 gene family is primarily involved in the JA, SA, ABA, and ethylene signaling pathways. Auxin is crucial for the morphogenesis of plants, including nutrient growth, root differentiation, vascular tissue differentiation, axillary bud differentiation, and flower organ formation [64]. The roles of *GH3* vary in different species, growth stages, and tissue types. In Arabidopsis, *AtGH3.3* and *AtGH3.5* played important roles in root development [28, 65]. Similar to these, *CsGH3.3*, *CsGH3.5*, *CsGH3.10*, and *CsGH3.16* in tea plants are mainly expressed in roots, suggesting the involvement of *GH3* in regulating root development in tea plants. Additionally, *CsGH3.3* and *CsGH3.10* are highly expressed in flower tissues of tea plants. In rice, *OsGH3.1*, *OsGH3.4*, *OsGH3.5*, *OsGH3.8*, and *OsGH3.11* exhibited the highest expression in flowers [29]. Moreover, as a target of the MADS-box, *OsGH3.8* is involved in the differentiation of floral organs in rice [66]. In chickpeas, *CaGH3.10* played an important role in regulating the steady state of auxin during early flower organ development [67]. These results suggested that *CsGH3* played an important role in both the growth and reproductive development of tea plants.

Through previous predictions, it was found that the promoter region of the *CsGH3* gene is enriched with several transcription factors. AP2/ERF transcription factors have been proven to participate in plant responses to abiotic stress [68]. Additionally, studies have shown that members of the ERF subfamily can bind to ethylene-responsive elements (EREs) in gene promoter regions, mediating ethylene biosynthesis [69]. SOC transcription factors, as important members of the MADS-box family, were widely involved in flower organ formation, development, and flowering time regulation [70]. Therefore, we speculated that the formation of the IAA-conjugate mediated by *CsGH3* is likely closely related to ethylene biosynthesis and may also be involved in the growth and development of flower organs. The expression of *GH3* genes is not only influenced by hormones and various biotic and abiotic stresses but also regulated by upstream transcription factors. Transcription factors such as ARF [71], bZIP [72], and R2R3-MYB [73] have been proven to regulate the expression of *GH3* genes by binding to *cis*-acting elements in the *GH3* gene promoter region. In this study, the potential regulatory role of the transcription factor CsDELLA on *CsGH3.14* and *CsGH3.15* was predicted and verified through yeast one-hybrid assays. DELLA proteins are essential components in the GA signaling pathway, and research has shown that environmental and hormonal signals, such as auxin and abscisic acid, can regulate plant growth by affecting the stability of DELLA proteins [74, 75]. Growth hormone enhances the instability of DELLA proteins, leading to the promotion of root growth. In poplar, AUXIN RESPONSE FACTOR 7 (ARF7) forms a ternary complex with Aux/INDOLE-3-ACETIC ACID 9 (IAA9) and DELLA, mediating cross-talk between the auxin and GA signaling pathways during cambium development [76]. In the growth and development processes of plants, multiple hormone signals and transcription factors form a complex and precise regulatory network, orchestrating various life processes in plants. This experiment, for the first time, identified the transcriptional regulation of key IAA metabolism gene *CsGH3* by the GA signaling-related factor CsDELLA in tea plants, providing new insights into the interplay between GA and auxin signaling in tea plants.

## Conclusion

It was deduced that the GH3 gene family plays important roles in plants. They are involved in hormone regulation, stress responses, and growth and development activities. Different *GH3* members exhibit regulatory mechanisms, with their expression being regulated by various hormones and external environmental factors. The promoter regions of *CsGH3* genes contain multiple *cis*-acting elements that can be regulated by transcription factors. In tea plants, *CsGH3* genes have undergone evolution and duplication events, leading to diverse gene compositions and regulatory patterns. Further research on the functions and regulatory mechanisms of the GH3 gene family is of great significance in unraveling the molecular mechanisms underlying plant growth, development, and responses to stress.

### Electronic supplementary material

Below is the link to the electronic supplementary material.

**Supplementary Material 1: Figure S1** (Validation of RNA in various tissue parts of tea plants), **S2** (Verification of plasmid construction in *E. coli* DH5α colonies using colony PCR), **S3** (Verification of plasmid construction in Yeast Y1H colonies using colony PCR (A, B)), and **S4** (Self-activation detection of *CsGH3.14* and *CsGH3.15*)

**Supplementary Material 2: Table S6**. Binding Site Prediction Results

## Data Availability

The datasets analysed and all the accession numbers of the sequences during the current study are provided in supplementary Tables S1 (Primer sequences used in PCR), S2 (Physicochemical properties of CsGH3), S3 (Evolutionary analysis of the CsGH3 gene family), S4 (Co-linear genes of GH3 in tea plants and Arabidopsis), S5 (Physical and chemical properties of the CsGH3 gene family), S6(Binding Site Prediction Results), S7 (Relative expression for CsGH3 in flower bud, flower,young leaf,old leaf, stem, and rootin Camellia sinensis using qRT-PCR), and S8 (Prediction results of screened binding siteswhich can also be downloaded fromTea Plant Information Archive (http://tpia.teaplants.cn/index.html) and Arabidopsis database (http://www.arabidopsis.org/).
