# Supplementary material for: Genome-wide identification and expression characterization of the GH3 gene family of tea plant (Camellia sinensis)
Source: BMC Genomics. 2024 Jan 27;25:120. doi: 10.1186/s12864-024-10004-y (PMC10822178; doi:10.1186/s12864-024-10004-y)
Supplement: Supplementary file 1 — Supplementary Material 1: Figure S1 (Validation of RNA in various tissue parts of tea plants), S2 (Verification of plasmid construction in E. coli DH5α colonies using colony PCR), S3 (Verification of plasmid construction in Yeast Y1H colonies using colony PCR (A, B)), and S4 (Self-activation detection of CsGH3.14 and CsGH3.15) [file 12864_2024_10004_MOESM1_ESM.docx]

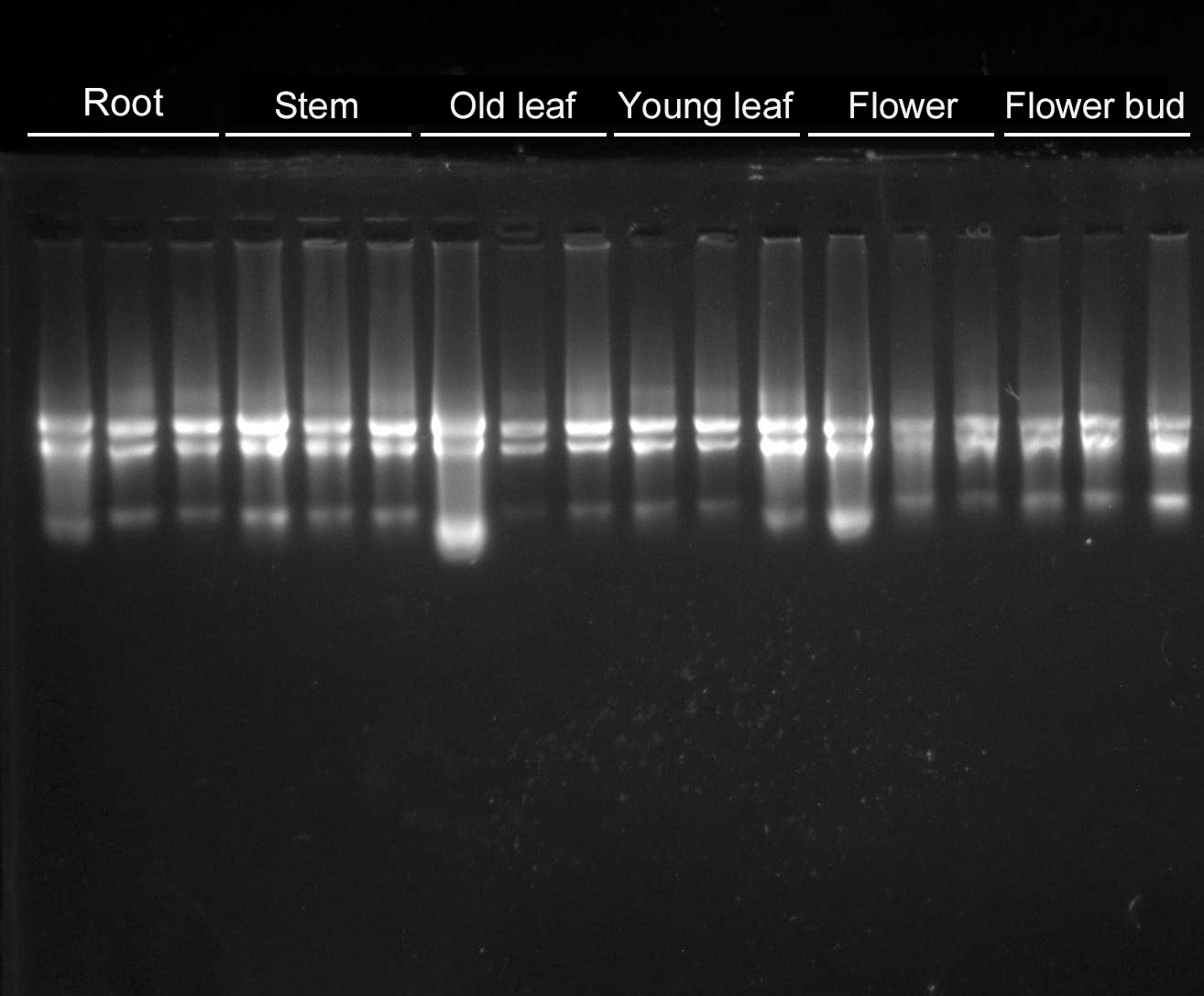


Figure S1 Validation of RNA in various tissue parts of tea plants.


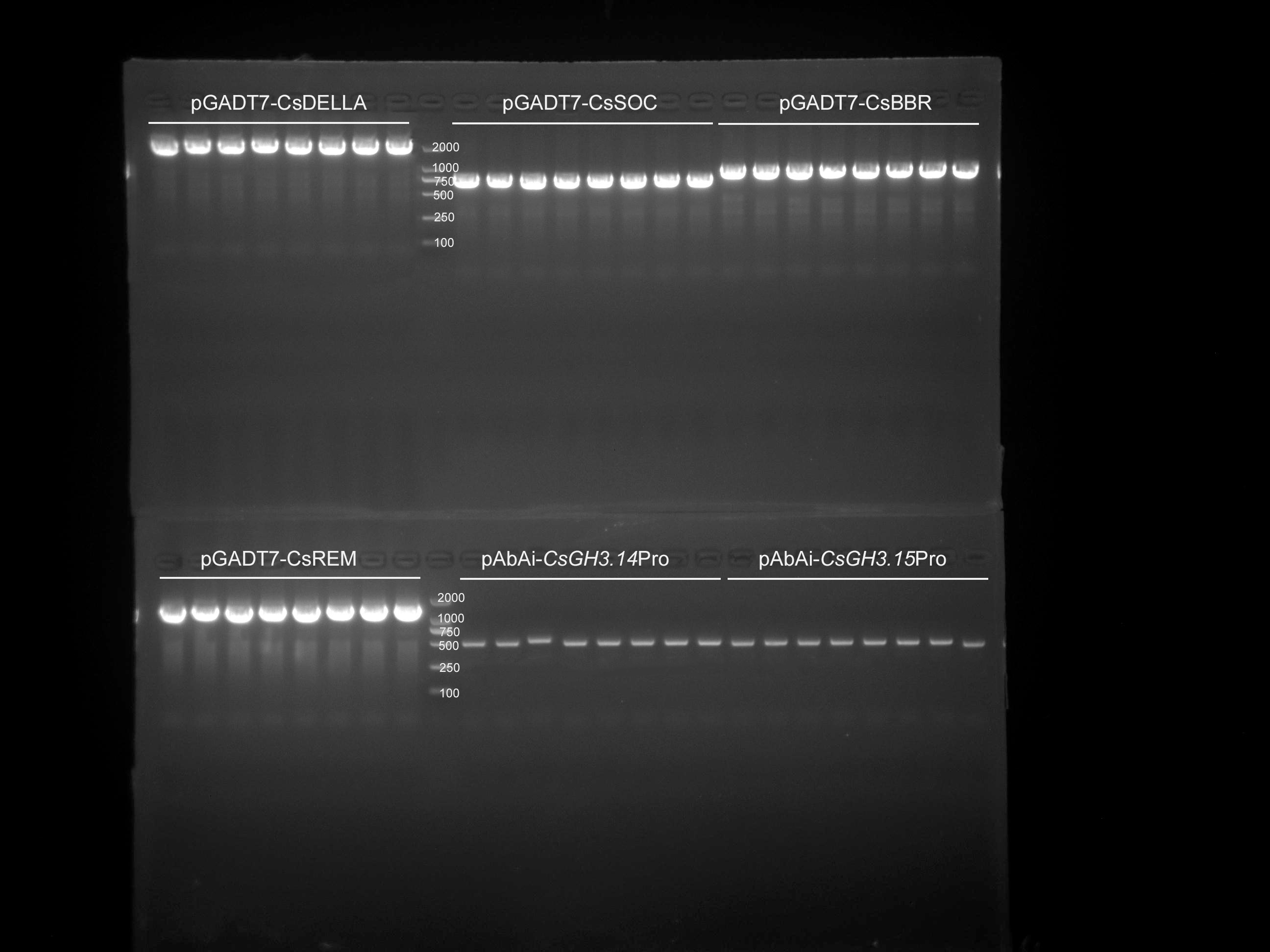


Figure S2 Verification of plasmid construction in *E. coli* DH5α colonies using colony PCR.

A


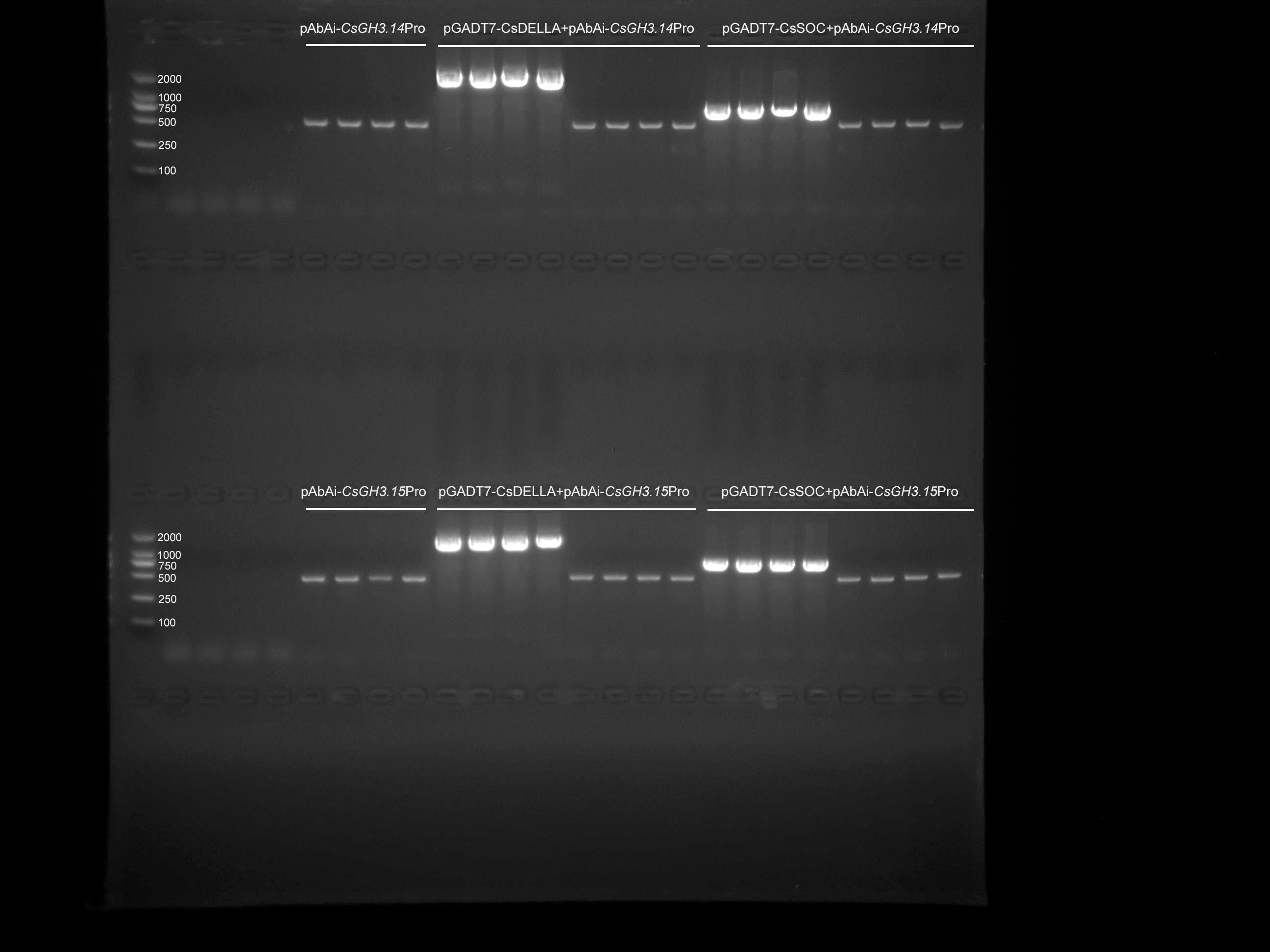


B


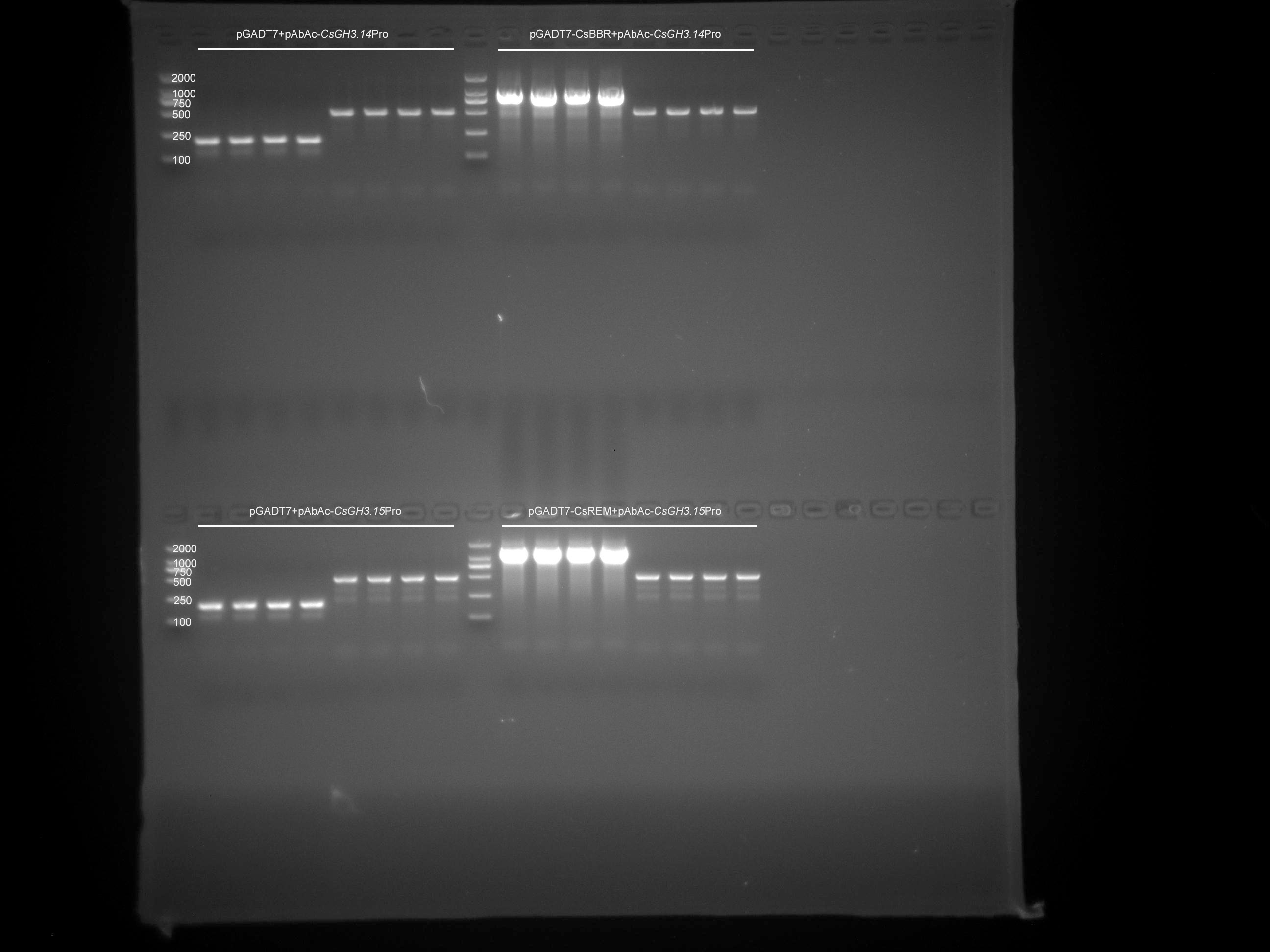


Figure S3 Verification of plasmid construction in Yeast Y1H colonies using colony PCR (A, B).


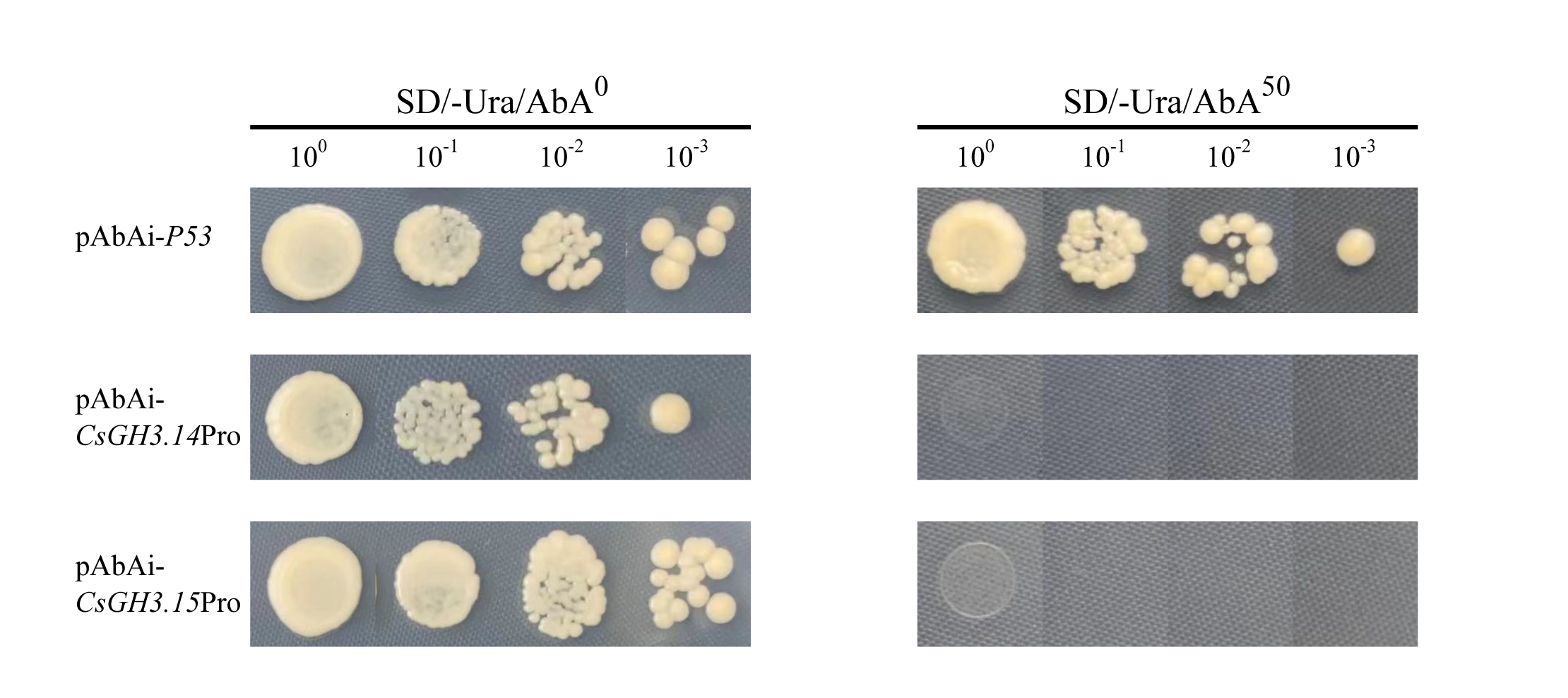


Figure S4 Self-activation detection of *CsGH3.14* and *CsGH3.15*.
